# Development of a Serious Game to Simulate Neonatal Intensive Care Unit Experiences: Collaborative Quasi-Experimental Study

**DOI:** 10.2196/73009

**Published:** 2025-06-18

**Authors:** Yukihide Miyosawa, Koichi Hirabayashi, Kodai Yamada, Fumiya Kobayashi, Nanami Ogihara, Noa Takeda, Eri Okamura, Shogo Matsumura

**Affiliations:** 1 Department of Pediatrics Shinshu University School of Medicine Matsumoto Japan; 2 Department of Medical Education Shinshu University School of Medicine Matsumoto Japan; 3 Department of Illustration Tokyo Designer Gakuin College Tokyo Japan; 4 Department of Medical Education Keio University School of Medicine Keio University Tokyo Japan

**Keywords:** collaborative development, medical education, neonatal care, neonatal intensive care unit, physicians and students, serious game, simulation

## Abstract

**Background:**

Opportunities for neonatal intensive care unit (NICU) training are limited for medical and nursing students due to patient safety concerns and the complexities of neonatal care. In addition, the COVID-19 pandemic significantly disrupted clinical training opportunities, further underscoring the need for alternative educational tools that can provide immersive and practical learning experiences. Serious games have garnered attention as potential tools for medical education; however, few are designed to simulate the complete NICU environment and its unique challenges.

**Objective:**

To address the educational gaps in neonatal care training, we aimed to develop and evaluate a serious game that provides a comprehensive NICU simulation experience for students and the general public.

**Methods:**

The game was developed over 14 months by a collaborative team that included a neonatologist, 4 medical students, and 1 art student, with a total cost of US $10,000. Initially created in TyranoBuilder (STRIKEWORKS), the game was later redeveloped in Unity with Naninovel to support multilingual functionality. Structured as a 6-chapter visual novel, the game follows a high school student observing the NICU during a hospital internship. Scenario-based decision-making and interactive dialogues guide the player through both the clinical and emotional aspects of neonatal care. After completing the game, players were invited to participate in an optional web-based survey that assessed demographic information, gameplay quality, and educational value using Likert scales. Descriptive and inferential statistics were used for data analysis.

**Results:**

The game, titled First Steps in the NICU, was released for iOS, Android, and Steam. As of May 2025, it has been downloaded 2799 times (2260 on iOS and 539 on Android). A total of 160 survey responses were collected, with 46.3% of respondents identifying as health care professionals or students. The majority of participants were female (114/160, 71.3%) and aged 20-29 years (59/160, 36.9%). Mean scores for length, difficulty, and gameplay were 3.05 (SD 0.62), 2.49 (SD 0.76), and 3.65 (SD 0.77), respectively, indicating a well-balanced design. The educational usefulness of the game received high ratings: empathy with the story (4.24), usefulness for knowledge acquisition (4.16), and effectiveness of serious games as a learning tool (4.37). No significant differences in evaluations were found between health care professionals and students and the general public, suggesting broad accessibility and appeal.

**Conclusions:**

We developed a low-cost serious game that simulates NICU experiences through collaboration between a neonatologist and students. The game received positive feedback and demonstrated educational value for a diverse audience. Positioned as formative research, this study highlights the potential of serious games to supplement neonatal care education. Future updates will incorporate user feedback, leading to improvements in gameplay and expanded content.

## Introduction

Serious games are designed to address social issues and are used across various fields, including education, government, and health care [1,2]. These games offer several benefits, such as increasing engagement, providing a safe environment to practice skills, and enabling personalized learning experiences [3,4].

In health care, serious games have been widely adopted as educational tools [5], with numerous reports highlighting their effectiveness in aiding the acquisition of diagnostic skills and surgical techniques [6]. However, their application in simulating immersive medical experiences across broader health care fields remains limited [7].

In neonatal care, serious games have primarily been implemented in neonatal resuscitation training, with many studies demonstrating their effectiveness in this area [8,9]. However, there are few reports on serious games that allow students to experience the full scope of neonatal intensive care unit (NICU) settings and the complexities of neonatal care. Premature and critically ill neonates in the NICU are at high risk of sudden deterioration, making it challenging for students to conduct examinations or perform medical procedures. For medical and nursing students, these high-stakes situations create substantial psychological barriers to engaging with neonatal care [10]. Furthermore, the COVID-19 pandemic significantly restricted clinical training opportunities, highlighting the potential for future pandemics to impose similar limitations, resulting in the loss of valuable learning experiences [11].

Based on the above considerations, we hypothesized that a serious game simulating a comprehensive NICU experience could serve as an effective tool to enhance medical and nursing students’ understanding of neonatal care. In 2021, we developed a serious game to simulate clinical training in the NICU. This project was undertaken solely by a team of a neonatologist, medical students, and a design student without involvement from professional game developers. This study outlines the development process of the serious game and evaluates its educational effectiveness based on player feedback. Therefore, the objective of this study was to investigate whether a collaboratively developed serious game could enhance users’ understanding of neonatal intensive care, particularly among medical and nursing students. We hypothesized that this game would provide educational value and promote engagement among a diverse audience, including health care professionals and the general public.

## Methods

### Preparation and Design

The game development spanned 14 months, from July 2021 to August 2022. The Serious Game Development Committee consisted of 6 members: 1 neonatologist who served as the project leader, 4 medical students, and 1 art student specializing in illustration. The game’s storyline was created by the neonatologist to accurately represent the NICU experience. Nurses, midwives, and obstetricians from Shinshu University Hospital contributed to the medical accuracy of the content through interviews. After finalizing the storyline, the team programmed the game. Programming was conducted by 4 medical students in collaboration with the neonatologist, while 1 student processed background images by converting photographs into illustrations to create a cohesive visual style. Character design was performed by the art student specializing in illustration. All students involved in this project were used as part-time assistants and met with the neonatologist in the laboratory after classes to work on the development of the game. After the prototype was completed, perinatal health care professionals tested the game. Their feedback on the accuracy of medical information and overall functionality was incorporated into the final version to ensure that it met educational and practical standards.

### Story

The game is structured as a visual novel, allowing players to follow a narrative-driven experience with interactive choices. Players assume the role of a high school student observing the NICU as part of a 1-day hospital internship with the goal of compiling a report based on their experience.

**Figure 1:**
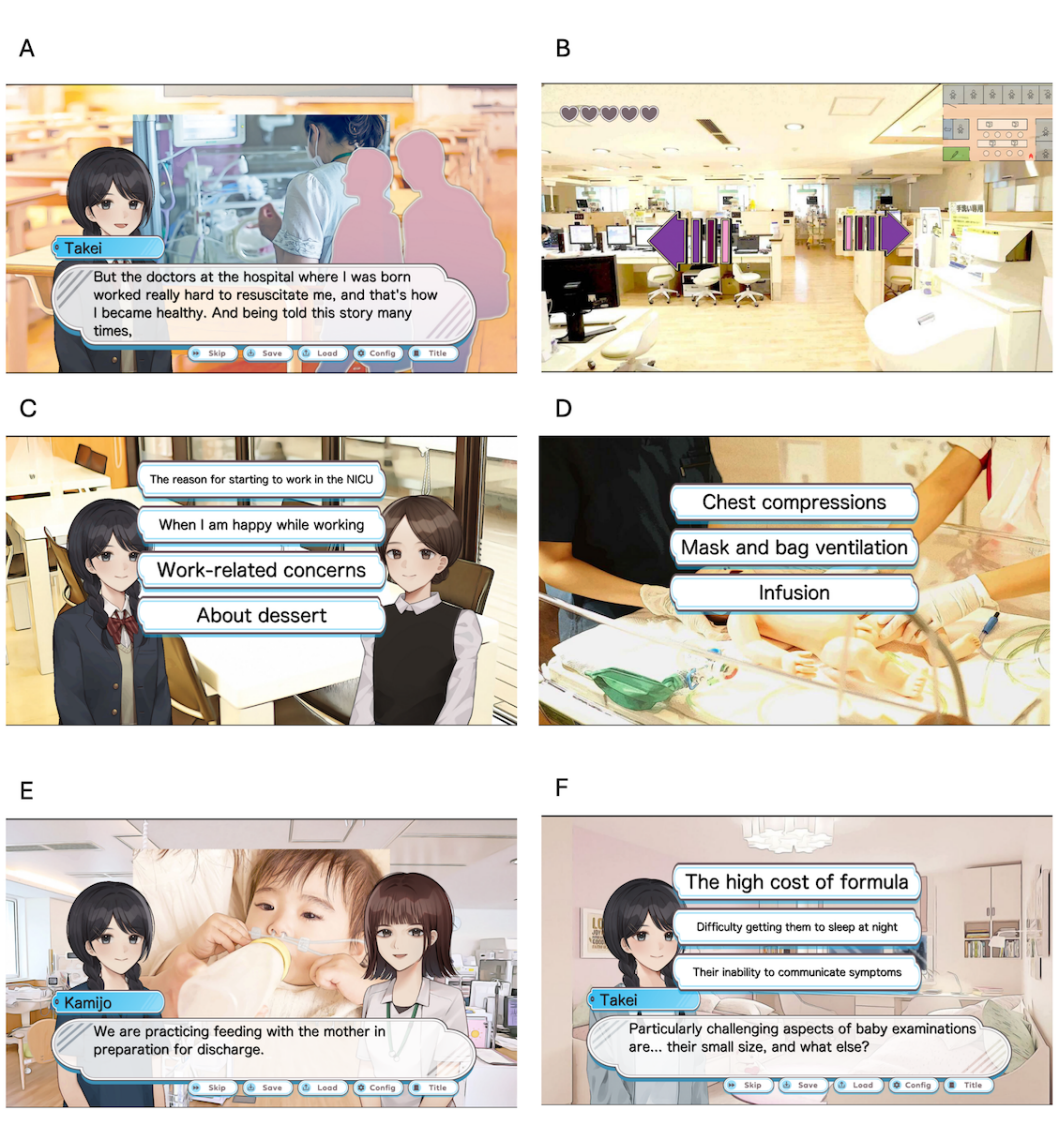
Overview of the neonatal intensive care unit simulation game stages. (A) High school scene: the protagonist, a high school student, expresses their interest in the neonatal intensive care unit to classmates and prepares a previsit report, setting the foundation for their upcoming hospital experience. (B) Neonatal intensive care unit exploration: the player freely explores the neonatal intensive care unit environment, learning about medical equipment such as ventilators and ultrasound machines, neonatal care practices, and the emotional perspectives of health care providers and families involved in neonatal care. (C) Lunch with staff: during a meal break with doctors and nurses, the player can engage in open conversations, gaining insights into the motivations and experiences of neonatal intensive care unit health care professionals. (D) Emergency scenario: the player participates in a simulated emergency response, providing an opportunity to learn about critical actions during neonatal resuscitation and acute care. (E) General care unit and discharge support: the player transitions to the general care unit to learn about discharge planning and the support necessary for families preparing to take their newborns home. (F) Report compilation: after completing the neonatal intensive care unit experience, the player consolidates their knowledge and reflections into a report, reinforcing the learning objectives from each stage of the simulation.

The game is divided into 6 chapters and offers a comprehensive NICU experience, gradually introducing players to the NICU environment and its unique challenges (Figure 1).

Throughout the game, players interact with doctors, nurses, and families, and their choices influence the storyline, leading to multiple possible outcomes. In the exploration phase, players navigate the NICU freely, examining various aspects of neonatal care. This includes interacting with medical equipment such as ventilators and ultrasound machines, learning emergency response protocols, and understanding family support systems. The estimated total playtime for the game is approximately 1 hour.

### Technology

The game was initially developed using TyranoBuilder (STRIKEWORKS), a game engine specialized for visual novels. TyranoBuilder’s visual scripting interface facilitates learning and development even for novice developers. This tool enables the integration of branching storylines, achievement tracking, and multiple endings. The game was first released as an iOS and Android application in September 2022. However, as of 2023, TyranoBuilder did not support multilingual functionality (a feature introduced in 2024). To provide multilingual versions in Japanese and English, the game was redeveloped in September 2023 using Naninovel, a visual novel engine for Unity (Naninovel, Elringus). Unity is a widely used game development platform and Naninovel simplifies the creation of interactive narratives within Unity. The multilingual version was successfully released in November 2024. The game, titled “First Steps in the NICU” in English, was made freely available for download on the Apple App and Google Play stores without restrictions. In addition to releasing the game on the App Store and Google Play, the game was also made available on Steam in February 2025, expanding its accessibility to a wider audience on PC platforms. This multiplatform approach was intended to reach a diverse demographic and enhance user engagement.

### Game Evaluation

After completing the game, players were invited to participate in an optional survey conducted via Google Forms. Textbox 1 outlines the survey questions.

The survey gathered demographic information such as occupation, age, gender, device used for the game, and playtime. To assess the game’s quality and educational value, the survey used a series of 5-point Likert scales listed in Textbox 2.

The scenes that left the strongest impressions and requests regarding topics for future games were also collected. Finally, participants were given the opportunity to provide additional insights through open-ended comments. We also retrieved data on user engagement from App Store Connect and Google Play Console. These include download trends over time and device usage distributions.

**The list of questions in the survey.**

Questions and their choices

1. What is your occupation?

- Pediatrician
- Physician other than pediatrician
- Nonphysician health care professional
- Medical student
- Nursing student
- Non–health care professional
- Non–health care university student
- Elementary, junior high, and high school student
- Other

2. What is your age? (years)

- 10-19
- 20-29
- 30-39
- 40-49
- 50-59
- 60+

3. What is your gender?

- Men
- Women
- Other

4. What device did you use?

- Smartphone
- Tablet
- Personal computer
- Other

5. How long did it take you to complete the game? (hours)

- <1
- 1-2
- 2-4
- >4

6. How did you find the game length?

- 1 (too short), 2, 3, 4, 5 (too long)

7. How did you find the game difficulty?

- 1 (too easy), 2, 3, 4, 5 (too hard)

8. How much gameplay should the game include?

- 1 (less gameplay), 2, 3, 4, 5 (more gameplay)

9. Could you empathize with the story?

- 1 (slightly empathetic), 2, 3, 4, 5 (very empathetic)

10. Was the game useful for knowledge acquisition?

- 1 (slightly useful), 2, 3, 4, 5 (very useful)

11. Do you think serious games are effective as a learning tool?

- 1 (slightly effective), 1, 2, 3, 4, 5 (very effective)

12. What scenes left the strongest impression on you? (Select all that apply.)

- From high school to neonatal intensive care unit (NICU) admission
- NICU exploration
- Learning how to care for the baby
- Chatting over lunch
- Baby’s hospitalization
- Discharge support at the general care unit
- Report writing to the ending

13. What topics would you like future serious games to cover? (select all that apply)

- Discharge support from the NICU
- Infant care institutions
- Disaster preparedness for children requiring medical care
- Home support for children requiring medical care
- Child development support facilities
- School attendance for children requiring medical care
- Employment support for children requiring medical care
- Using administrative services for children requiring medical care
- Other

14. Please feel free to share any additional comments or feedback.

**Likert scales for game quality and educational usefulness matrices.**

**Game quality metrics**

- Length: 1 (too short) to 5 (too long).
- Difficulty: 1 (too easy) to 5 (too hard).
- Gameplay: 1 (needs less gameplay) to 5 (needs more gameplay).

**Educational usefulness metrics**

- Empathy with the story: 1 (slightly empathetic) to 5 (very empathetic).
- Usefulness for knowledge acquisition: 1 (slightly useful) to 5 (very useful).
- Effectiveness of serious games for learning: 1 (slightly effective) to 5 (very effective).

### Statistical Analyses

Participants’ characteristics were summarized using descriptive statistics. A comparison of the results between the 2 groups was performed using unpaired *t* tests for continuous variables and Fisher exact tests for categorical variables. Graph generation and statistical analyses were performed using GraphPad Prism version 9 (GraphPad Software) or EZR (Saitama Medical Center, Jichi Medical University) software [12]. Statistical significance was set at *P*<.05 (2-sided).

### Ethical Considerations

This study was approved by the Ethics Committee of Shinshu University (approval number 5794) on March 14, 2023. The research protocol was titled “Educational Effects of Experiencing Neonatal Medical Care Through a Serious Game.” Participation in the postgame survey was voluntary, and informed consent was obtained through a statement provided at the beginning of the questionnaire. The survey was anonymous, and no personally identifiable information was collected. Participants did not receive any compensation for their participation.

## Results

### Overview of Downloads by Device Type, Operating System, and Country

The game has been downloaded 2260 times on iOS and 539 times on Android as of May 2025. In terms of device type, the majority of downloads were from smartphones (2540/2796, 90.8%), followed by tablets (230/2796, 8.2%) and other devices (29/2796, 1%; Table S1 in Multimedia Appendix 1). Geographically, Japan accounted for the overwhelming majority of downloads (2703/2796, 96.6%), reflecting the fact that the Japanese version was the primary distribution channel at the time of data collection. Notably, the application was also accessed by users in North America, South and Southeast Asia, and the Middle East, demonstrating its potential international reach (Table S2 in Multimedia Appendix 1).

### Overall Respondent Characteristics and Game Evaluations

Responses were collected from 160 participants. Table 1 presents the respondents’ characteristics. Health care professionals and students comprised 74 out of 160 (46.3%) participants, while the general public accounted for 86 out of 160 (53.8%) participants. The highest proportion of respondents was in the 20- to 29-year age range, accounting for 59 out of 160 (36.9%) participants, with 114 out of 160 (71.3%) participants identifying as female.

**Table 1:** Characteristics of survey respondents (N=160).

Characteristics			Values, n (%)
**Occupation**			
	Pediatricians	10 (6.3)	
	Physicians other than pediatricians	3 (1.9)	
	Nonphysician health care professionals	31 (19.4)	
	Medical students	18 (11.3)	
	Nursing students	12 (7.5)	
	Non–health care professionals	44 (27.5)	
	Non–health care university students	6 (3.7)	
	Elementary, junior high, and high school students	24 (15)	
	Other	12 (7.5)	
**Age group (years)**			
	10-19	25 (15.6)	
	20-29	59 (36.9)	
	30-39	38 (23.7)	
	40-49	26 (16.2)	
	50-59	10 (6.3)	
	60+	2 (1.2)	
**Gender**			
	Men	46 (28.7)	
	Women	114 (71.3)	
	Other	0 (0)	
**Playtime (hours)**			
	<1	99 (61.9)	
	1-2	53 (33.1)	
	2-4	3 (1.9)	
	>4	5 (3.1)	

**Figure 2:**
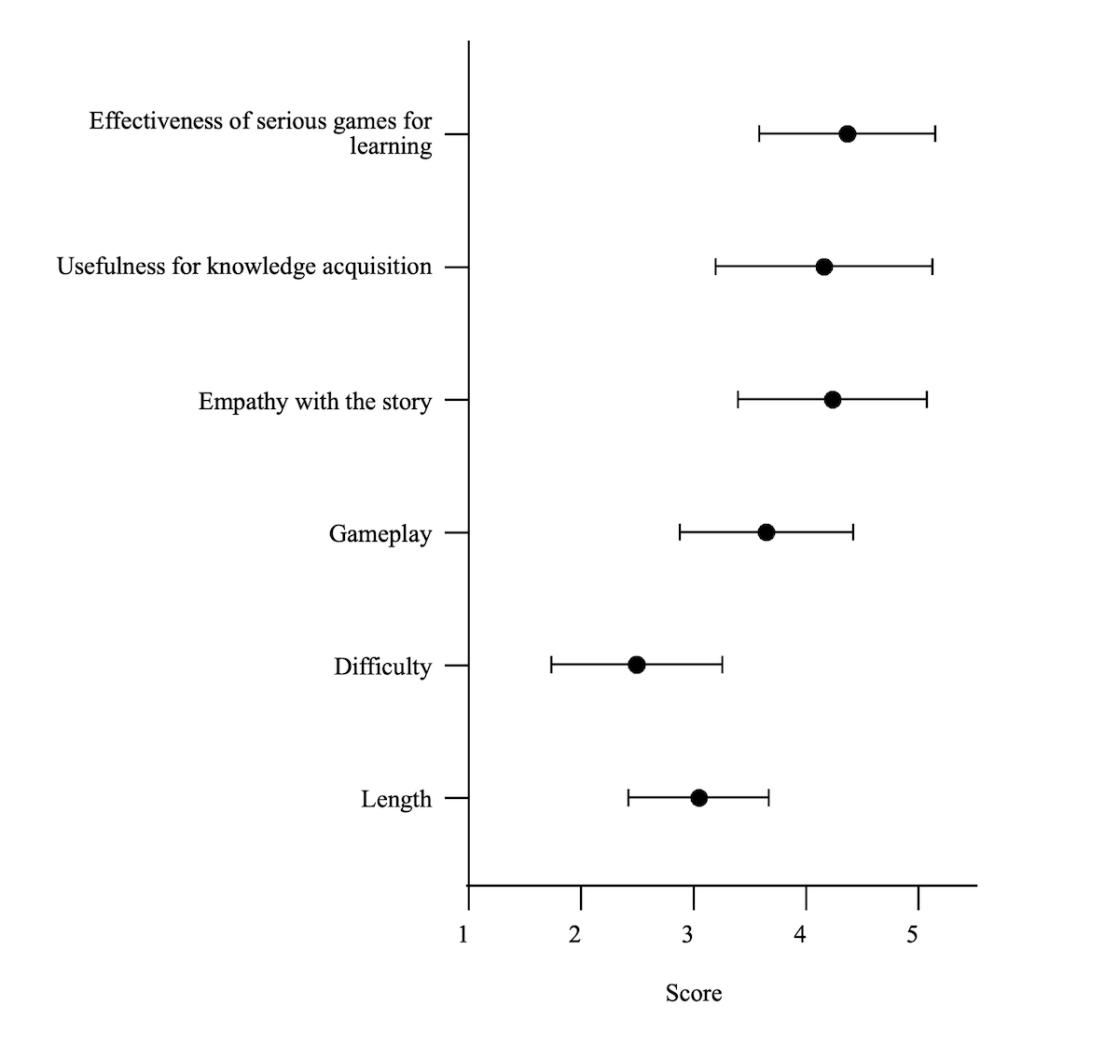
Game evaluations in overall respondents The respondents rated six aspects of the game: length, difficulty, gameplay, empathy with the story, usefulness for knowledge acquisition, and effectiveness of serious games for learning. The black spots and error bars indicate the mean and standard error, respectively.

The mean scores for length, difficulty, gameplay, story empathy, usefulness for knowledge acquisition, and effectiveness of serious games for learning were 3.05 (SD 0.62), 2.49 (SD 0.76), 3.65 (SD 0.77), 4.24 (SD 0.84), 4.16 (SD 0.96), and 4.37 (SD 0.78), respectively (Figure 2).

### Comparison Between the General Public and Health Care Professionals and Students

To assess the differences in ratings for the serious game between the general public and health care professionals and students, we compared the questionnaire responses. While there were no differences in age or gender, playtime was significantly shorter among health care professionals and students than among the general public (Table 2).

**Figure 3:**
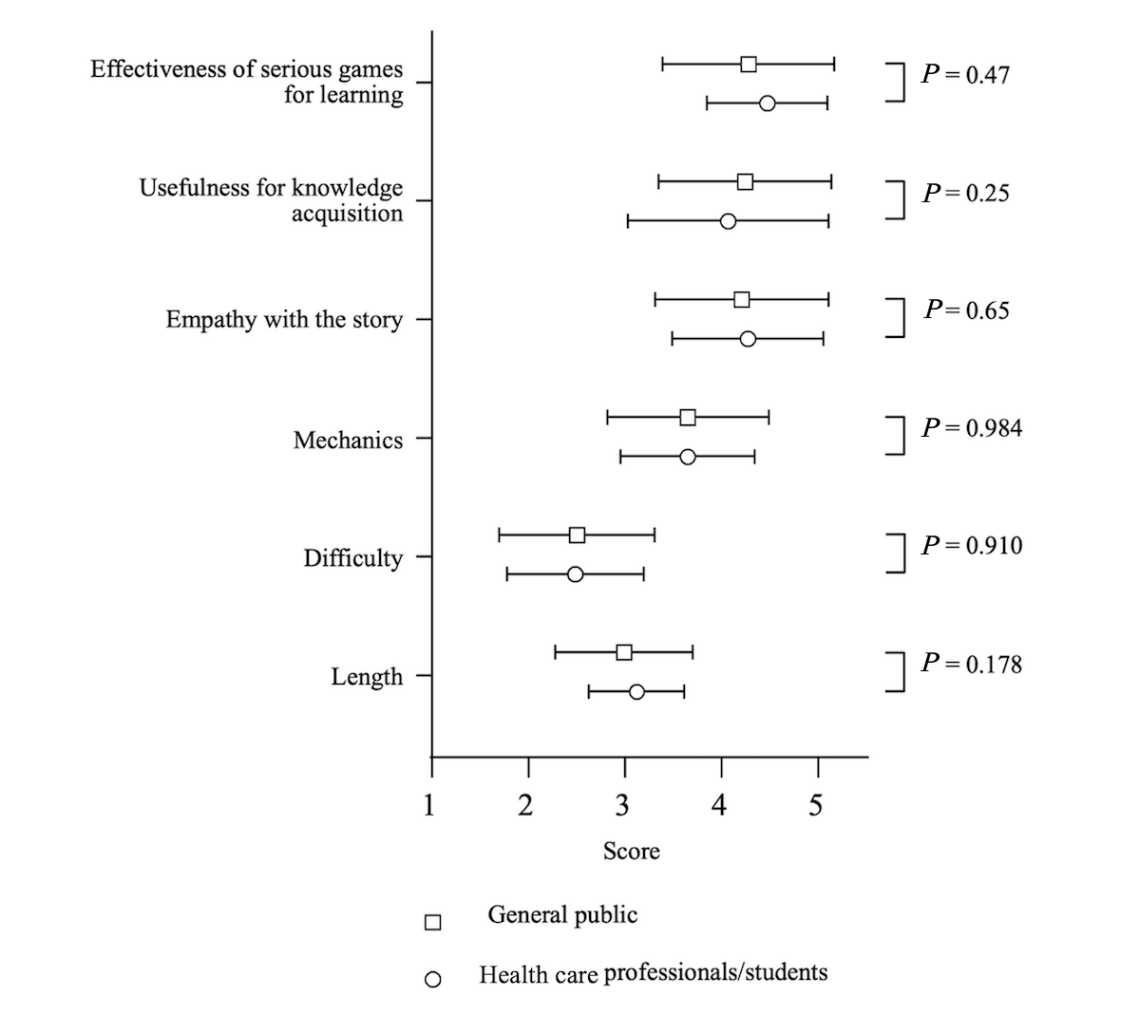
Comparison of game evaluations between health care professionals and students and the general public. The mean ratings of the game evaluations by the general public and health care professionals and students are shown as open squares and circles, respectively. The error bars indicate the SE.

There were no differences in the ratings for the 6 questions between the 2 groups (Figure 3).

**Table 2:** Comparison of the characteristics of health care professionals and students and the general public.

Characteristics		Health care professionals and students (n=74), n	General public (n=86), n	*P* value	
**Age group (years)**					.75
	10-19	1	24		
	20-29	41	18		
	30-39	15	23		
	40-49	15	11		
	50-59	2	8		
	60+	0	2		
**Gender**					.49
	Male	19	27		
	Female	55	59		
**Playtime**					.03
	Less than 1 h	53	46		
	1-2 h	19	34		
	2-4 h	0	3		
	More than 4 h	2	3		

## Discussion

### Main Findings

This study demonstrated that a serious game simulating the NICU experience, developed collaboratively by a neonatologist and university students, was positively evaluated by both health care professionals and the general public. The game was found to offer high educational value and accessibility across diverse user groups.

### Development Process and Cost Efficiency

A serious game designed to simulate clinical training in the NICU for medical and nursing students was created by a team comprising a neonatologist, medical students, and a design student. Unlike existing tools that focus on specific skills, such as neonatal resuscitation [8,9], this game offers a broader educational experience covering various aspects of neonatal medicine, making it a unique resource in medical education.

A major concern regarding the development of serious games among health care professionals is the lack of technical expertise in programming and limited funding [13,14]. In this study, the game was developed without the involvement of professional game developers. Despite a limited budget of approximately 1.5 million yen (US $10,000) for this project, a serious game was successfully created using TyranoBuilder, a low-cost visual novel development tool with an easy learning curve. This tool enabled medical students with no prior programming experience to contribute directly to the creation of the game. In addition to reducing costs, this approach likely helped students gain a deeper understanding of neonatal care. The art student’s participation in a socially impactful project may have also enhanced their confidence, emphasizing the interdisciplinary value of educational game development. Our serious game development process demonstrates that health care professionals without technical programming expertise can create serious games, suggesting that these games can be developed at low costs.

### Game Design and Educational Value

Balancing game design is crucial for maximizing both game engagement and educational value. In this study, we assessed the balance of the serious game using 3 parameters: length, difficulty, and gameplay. On the 5-point Likert scale, a score of 3 indicates an ideal rating. The mean scores for length, difficulty, and gameplay were 3.05, 2.49, and 3.65, respectively, indicating a balanced design. However, to improve the experience, increasing both the difficulty and gameplay could be ideal for future versions.

The main purpose of the serious game was to educate medical and nursing students. We assessed the educational usefulness of our serious game using 3 parameters: empathy with the story, usefulness for knowledge acquisition, and effectiveness of serious games for learning. The mean scores for all 3 parameters exceeded 4 on a 5-point Likert scale (Figure 3), suggesting that our serious game offers substantial educational value. Although previous studies have criticized serious games for offering lower levels of enjoyment compared to commercial video games [15], our findings suggest that this game may overcome this problem. These findings suggest that serious games have the potential to serve as educational tools and models for future game development in medical education. These findings can also be understood through educational theory. Kolb experiential learning model emphasizes learning through active experience and reflection, which are incorporated into this game through scenario-based choices and integrated quizzes [16]. In particular, the 4 stages of the Kolb model (concrete experience, reflective observation, abstract conceptualization, and active experimentation) are represented, respectively, through the immersive NICU simulation, narrative reflection points, in-game medical content, and decision-making paths. This structure helps players internalize neonatal care concepts, potentially leading to real-world applications. Similarly, the game aligns with Bloom taxonomy by promoting not only memorization but also higher-order thinking skills such as application and analysis, suggesting it may be more effective than traditional knowledge-based education [17]. By integrating these learning theories into game design, the educational impact of serious games can be theoretically grounded and systematically enhanced, supporting their use in structured medical curricula.

### Comparison Between Health Care Professionals and the Public

We also compared the game’s ratings between health care professionals and students and the general public. Because the NICU is an unfamiliar and highly specialized environment, we anticipated lower ratings from the general public regarding balance and educational value. Contrary to our expectations, no significant differences were observed between the 2 groups. This indicates that our serious game is suitable for both health care professionals and students and the general public, providing NICU learning and immersive experiences.

Although the primary target of this serious game was medical and nursing students, a wide variety of people played the game and participated in the survey. Teenagers accounted for 25 out of 160 (15.6%) respondents. This is particularly important, as early exposure to medical concepts may inspire future interest in health care careers. The game’s accessible design makes it appealing to the general public, suggesting that increased outreach to younger demographics could be beneficial.

### Formative Nature and Future Development

This study can be positioned as formative research, as it focuses on the early-stage development and evaluation of an innovative educational tool. The postgame survey revealed that many participants provided positive feedback regarding the educational content and emotional impact of the game. However, some respondents expressed a desire for enhanced gameplay features and increased entertainment value. Based on these insights, future iterations of the game will explore ways to integrate more engaging mechanics while preserving educational depth. The authors are also involved in the ongoing development of serious games related to perinatal care and pediatric home medical support. In future projects, they aim to create tools that are both informative and enjoyable, appealing to a broader audience beyond health care professionals.

### Limitations and Future Directions

This study has several limitations. First, participation in the survey was voluntary and limited to players of the Japanese version of the game, potentially limiting the generalizability of the findings. In addition, the absence of a defined sampling frame and randomization process limits our ability to assess how representative the respondent population is. Second, the lack of an advertising budget may have reduced the diversity of respondents, potentially overrepresenting individuals connected to the Serious Game Development Committee. Moreover, the absence of a pretest and posttest design limits our ability to assess learning gains objectively. Consequently, the findings should be interpreted as preliminary and exploratory. The voluntary nature of the survey may also have introduced self-selection bias, potentially influencing the generalizability of the findings. Furthermore, the survey instrument was not formally validated or psychometrically tested and its reliability remains unverified. This lack of validation may affect the accuracy and consistency of the responses. Future studies should evaluate the multilingual version of the game and explore its impact across broader demographics and cultural contexts.

### Conclusions

We developed a serious game designed to enhance neonatal care education at a low cost, relying solely on the collaboration between a neonatologist and students. This serious game offers a comprehensive educational experience accessible to both health care professionals and students and the general public.

## Appendix

Multimedia Appendix 1 Overview of download by device type, operating system, and country.

## Abbreviations

NICU: neonatal intensive care unit
