# Supplementary material for: Development of a Serious Game to Simulate Neonatal Intensive Care Unit Experiences: Collaborative Quasi-Experimental Study
Source: JMIR Form Res. 2025 Jun 18;9:e73009. doi: 10.2196/73009 (PMC12223455; doi:10.2196/73009)
Supplement: Multimedia Appendix 1 [file formative_v9i1e73009_app1.docx]

**Table S1.** Download counts by device type and operating system.

| Device type | iOS (N) | Android (N) | Total (N) |
| --- | --- | --- | --- |
| Smartphone | 2,029 | 511 | 2,540 |
| Tablet | 208 | 22 | 230 |
| Other | 23 | 6 | 29 |
| Total | 2,260 | 539 | 2,799 |

**Table S2.** Download counts by country and operating system.

| Country | iOS (N) | Android (N) | Total (N) |
| --- | --- | --- | --- |
| Japan | 2,184 | 519 | 2,703 |
| United States | 29 | 3 | 32 |
| Canada | 8 | 1 | 9 |
| Saudi Arabia | 6 | 2 | 8 |
| India | 4 | 2 | 6 |
| Cambodia | 4 | 0 | 4 |
| Romania | 0 | 3 | 3 |
| Ethiopia | 0 | 3 | 3 |
| Others | 25 | 6 | 31 |
| Total | 2,260 | 539 | 2,799 |
